# Internet Addiction Through the Phase of Adolescence: A Questionnaire Study

**DOI:** 10.2196/mental.5537

**Published:** 2017-04-03

**Authors:** Silvana Karacic, Stjepan Oreskovic

**Affiliations:** ^1^ Health Center Sveti Kriz Trogir - Arbanija Croatia

**Keywords:** adolescents, Internet addiction, stages of adolescence

## Abstract

**Background:**

Adolescents increasingly use the Internet for communication, education, entertainment, and other purposes in varying degrees. Given their vulnerable age, they may be prone to Internet addiction.

**Objective:**

Our aim was to identify possible differences in the purpose of Internet use among adolescents with respect to age subgroup, country of residence, and gender and the distribution of Internet addiction across age subgroups. Another aim was to determine if there is a correlation between the purpose of Internet use and age and if this interaction influences the level of addiction to the Internet.

**Methods:**

The study included a simple random sample of 1078 adolescents—534 boys and 525 girls—aged 11-18 years attending elementary and grammar schools in Croatia, Finland, and Poland. Adolescents were asked to complete an anonymous questionnaire and provide data on age, gender, country of residence, and purpose of Internet use (ie, school/work or entertainment). Collected data were analyzed with the chi-square test for correlations.

**Results:**

Adolescents mostly used the Internet for entertainment (905/1078, 84.00%). More female than male adolescents used it for school/work (105/525, 20.0% vs 64/534, 12.0%, respectively). Internet for the purpose of school/work was mostly used by Polish adolescents (71/296, 24.0%), followed by Croatian (78/486, 16.0%) and Finnish (24/296, 8.0%) adolescents. The level of Internet addiction was the highest among the 15-16-year-old age subgroup and was lowest in the 11-12-year-old age subgroup. There was a weak but positive correlation between Internet addiction and age subgroup (*P*=.004). Male adolescents mostly contributed to the correlation between the age subgroup and level of addiction to the Internet (*P*=.001).

**Conclusions:**

Adolescents aged 15-16 years, especially male adolescents, are the most prone to the development of Internet addiction, whereas adolescents aged 11-12 years show the lowest level of Internet addiction.

## Introduction

Adolescence can be defined as the period between puberty and adulthood, usually between the ages of 11 and 18 years. Events during this period greatly influence a person's development and can determine their attitudes and behavior in later life [1]. Adolescence can be divided into three substages: early, middle, and late [2]. One of the most important functions of adolescence is to find one's own identity and view of life, without inner conflict and the need to always act within acceptable moral standards, abide by parental authority, or meet peer expectations [3]. Because teenagers are often in conflict with authority and cultural and moral norms of society, certain developmental effects can trigger a series of defense mechanisms [2]. During adolescence, there is an increased risk of emotional crises, often accompanied by mood changes and periods of anxiety and depressive behavior, which adolescents attempt to fight through withdrawal, avoidance of any extensive social contact, aggressive reactions, and addictive behavior [4,5]. Adolescents are exceptionally vulnerable and receptive during this period and can become drawn to the Internet as a form of release. Over time, this can lead to an addiction. Adolescents are especially attracted to new technological methods of communication, which offer interaction with others and at the same time provide anonymity, impression of belonging to a community, and a sense of social acceptability. The Internet as a global network connects millions of people throughout the world and enables users to exchange information, which remains available at any time and any place [6]. However, as the most popular and sophisticated mass communication medium today, the Internet also poses perils for the children using the Internet without adult control, especially for the adolescents with free access to the content that is inappropriate for their age and stage of development [7]. Unlimited access to information on the Internet can be a source of amusement and generator of new interests [8], but it can also be a source of new and unknown threats. Recently, Internet addiction has attracted great interest from the public and scientists alike [9]. Some authors point out that excessive Internet use leads to social isolation [10]. Other authors emphasize the physical aspects of addiction [11,12], while others underscore psychological signs and symptoms indicative of Internet addiction [11,13,14]. An addiction is often the result of social crisis, lack of self-confidence, a need to conform, boredom, and the availability of an interesting and amusing pastime [15]. Scientists still debate whether Internet addiction should be included in the Diagnostic and Statistical Manual of Mental Disorders (DSM). In the fifth edition of the DSM, Internet addiction is equated with the addiction to Internet games [16]. However, the scientific community has not yet reached a consensus on whether Internet addiction and addiction to Internet games should be viewed together or separately. Some research shows that they should be viewed as separate entities [17,18]. Griffiths strongly argues that there is a fundamental difference between Internet addiction and addiction to the Internet. He claims that most people who are considered to be addicted to the Internet are Internet addicts (ie, they use the Internet as a tool for other addictions) [16,19,20]. A recent study pointed out numerous advantages of Internet use for students, such as wide access to literature, e-learning, online courses, and webinars [21]. However, frequent visits to websites such as online chat rooms, game websites, and similar sites can easily cause addiction and negatively impact student health and standards of learning. Children easily abandon traditional pastimes and replace them with time spent surfing the Internet. This can lead to late bedtime with subsequent sleep loss. In addition, children often consider life without the Internet boring, which can lead to a strong feeling of loneliness [21]. Adolescents need time to solve identity crises, affirm their attitudes, and establish social links and professional aims [22]. A previous study revealed that university students showed varying degrees of Internet addiction, psychological distress, and depression with respect to sex, age, year of study, and residential status [23]. Adolescents are most open to the various addictive temptations that the Internet presents during the period of adjustment. Emotional intelligence as a mediator can influence the degree of Internet addiction and predict such kind of activity [24]. Adolescents tend to be more prone to risky behavior and can indulge in addictive practices in order to cope with anxiety, frustration, and failure or because of the need for excitement, unrealistic optimism in relation to the feeling of invulnerability, or even the need to achieve their goals as a part of their transition into adult age [25]. The overuse of the Internet in this age group may be considered a risky behavior because it may lead to Internet addiction. The objectives of our study were to identify possible differences in the purpose of Internet use among adolescents with respect to age subgroup, country of residence, and gender and to determine the distribution of Internet addiction across different adolescent age subgroups. We tested the hypothesis that there is a correlation between the purpose of Internet use and age and that their interaction influences the level of addiction to the Internet.

## Methods

### Sample Selection

Study participants were 11- to 18-year-old students who attended regular public schools. They comprised a simple random representative sample of adolescents. Using a table of random samples, we selected the city of Split in Croatia, the town of Pakość in Poland, and the city of Turku in Finland. To select schools, we used the method of random numbers, always respecting the structure of education. In Split, four schools per 100 students were randomly selected, while two schools per 100 students were randomly selected in each of Pakość and Turku (see Table 1). All randomly selected schools had access to the Internet.

**Table 1:** Elementary and high schools in Croatia, Poland, and Finland randomly selected for the study.

School location	School name and address
**Split, Croatia**	
	Poljišani Elementary School, Viška 12
	Blatine-Škrape Elementary School, Kržice 2
	Third Gymnasium *S* chool, Matice Hrvatske 11
	First Gymnasium School, Teslina 10
**Pakość, Poland**	
	Szkoła Podstawową im. Powstańców Wielkopolskich w Pakości, Błonie 2
	Gimnazjum im.Ewarysta Estkowskiego w Pakości, ul.Szkolna 44
**Turku, Finland**	
	Raunistulan koulu, Teräsrautelan koulu /Suikkilan yksikkö, Talinkorventie 16
	Turun suomalaisen yhteiskoulun lukio, Kauppiaskatu 17

The study was approved by respective ministries of education in Croatia, Poland, and Finland and ethics committees of the schools. The schools were sent an invitation to participate in the study, with the assurance of complete student privacy protection. Informed consent was obtained from each student and their parents or guardians.

### Survey Approach

The questionnaire was developed in Google Docs format and sent to schools in an electronic form along with instructions and contact information of the researchers. The title page instructed the students to fill out the questionnaire fully and truthfully. They were informed that their participation was anonymous and voluntary and that the data would be used for research purposes only. General and particular importance of the research was also explained. Furthermore, the students were notified of the type and duration of the procedures used and informed of the confidentiality of the data gathered and the protection of privacy of the participants. The students were free to refuse participation or to withdraw from the study at any time without explanation. Having been notified of all the particulars, they proceeded to fill out the questionnaire.

Our research systematically analyzed all significant variables necessary for scientific research. The survey consisted of three parts. A standardized procedure of double translation was applied to each part for each country and its language. The initial step was defined by taking the general data and demographic measures. Demographic parameters used in the research included age, gender (1=female, 2=male), country of residence, and purpose of Internet use (eg, school/work or entertainment). Participants were asked to appraise whether they use the Internet more often for entertainment or educational purposes.

### Internet Addiction Questionnaire

Specific aspects that have been included in a more detailed analysis of research on Internet addiction have been assessed in previous studies. These include assessing Internet addiction using Young’s Internet Addiction Test (IAT) [26], also known as Young’s Internet Addiction Scale. The scale contains 20 questions based on the criteria for pathological gambling. These questions reflect typical addictive behavior. The scale reflects six dimensions of Internet addiction: salient preoccupation, overuse of the Internet, neglecting work responsibilities, expectation, lack of self-control, and neglecting social life. The authors have found that the factors of saliency and overuse are connected with a more intensive use, while neglecting work is only correlated with age and in a negative way. The conclusion drawn by the authors is that the IAT “does measure some of the key aspects of Internet addiction” [27]. In our study, the level of addiction was rated on a scale ranging from 20 to 100—normal (20-49), moderate addiction (50-79), and serious addiction (80-100). A 5-point Likert scale was used and answers to each question resulted in a score from 1 to 5 points—very rarely (1), rarely (2), often (3), very often (4), and always (5). The scale demonstrated excellent internal consistency with an alpha coefficient of .93 in this study.

### Statistical Analysis

For statistical analysis, SPSS Statistics for Windows version 17.0 (SPSS Inc) was used. For data analysis, three groups of methods were used: descriptive statistical analysis (ie, relative numbers, median, and measures of dispersion), inferential statistical analysis (ie, chi-square test, *t* test, and analysis of variance), and multivariate analysis (ie, reliability factor analysis). Correlations were tested at different levels of significance (ie, 5%, 1%, and 0.1%).

**Figure 1:**
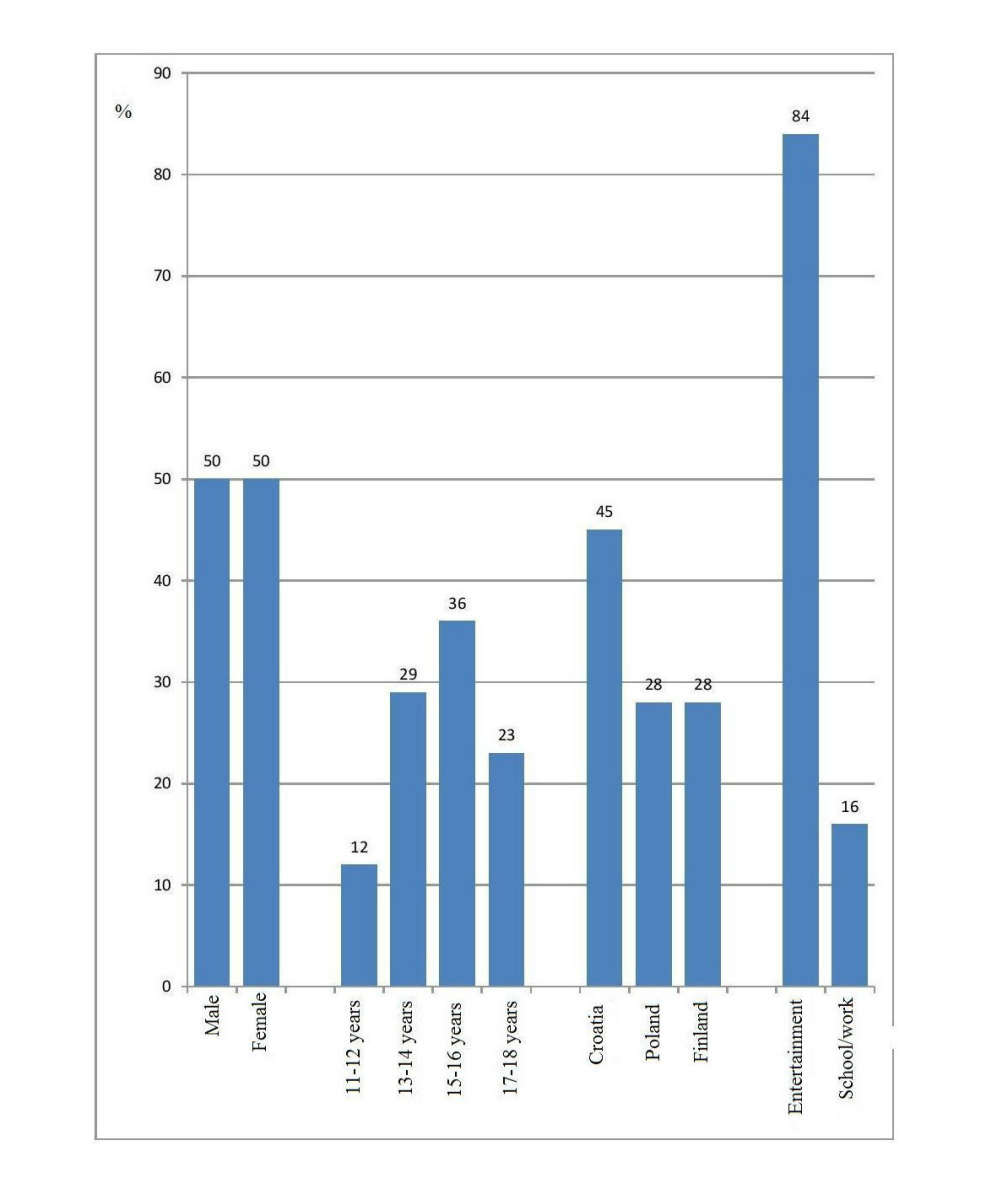
Distribution of adolescents (pooled data) with respect to gender, age, country of residence, and purpose of Internet use (in percentages).

## Results

### The Purpose of Using the Internet

A total of 1078 adolescents from Croatia, Poland, and Finland participated in the research (see Figure 1).

The representative sample included 534 boys and 525 girls; gender was unknown for 19 participants. The average age of participants was 14.9 years (SD 1.9, range 11-18), with an average discrepancy of 1.9 years, which is a small dispersion (variance coefficient 13%). Since participants were predominantly 15-year-olds, both the median and mode values were 15 years. There were more participants from Croatia than from either of the other two countries. A large majority of participants used the Internet for entertainment purposes (905/1078, 84.00%), whereas only a small proportion used it for school/work (172/1078, 16.00%).

### Internet Use and Gender

We found a statistically significant relationship between the purpose of Internet use and gender (χ^2^_1_=11.3; n=1042; *P*=.001) (see Table 2).

Female participants used the Internet for school- or work-related purposes (105/525, 20.0%) much more than their male counterparts (64/534, 12.0%). However, when the analyses of this correlation between gender and the purpose of Internet use were carried out separately for each country, the results became more revealing (see Table 3).

A statistically significant relationship was found for Croatian participants (χ^2^_1_=26.8; n=476; *P*<.001), but not for the Finnish (χ^2^_1_=0.2; n=275; *P*=.63) and Polish (χ^2^_1_=0.2; n=291; *P*=.65) participants. Therefore, the Croatian participants contributed to the statistically significant correlation between gender and the purpose of Internet use for the entire sample.

### Internet Use and Country of Residence

We found a statistically significant correlation between the country of residence and the purpose of Internet use (χ^2^_2_= 27.3; n=1048; *P*<.001) (see Table 4). The use of the Internet for school/work was less frequent among Croatian (78/486, 16.0%) participants than among Polish (71/296, 24.0%) participants, and it was only 8.0% (24/296) among Finnish participants.

**Table 2:** Number of interviewed adolescent participants according to gender and purpose of Internet use (n=1042).

		Purpose of Internet use, n		
		Entertainment	School/work	Total
**Gender**				
	Male	459	65	524
	Female	414	104	518
Total		873	169	1042

**Table 3:** Correlation between gender and purpose of Internet use for each country.

Country	χ^2^	df	n	*P*
Croatia	26.8	1	476	<.001
Poland	0.2	1	291	.65
Finland	0.2	1	275	.63

**Table 4:** Number of interviewed adolescent participants according to their country of residence and purpose of Internet use (n=1048).

		Purpose of Internet use, n		
		Entertainment	School/work	Total
**Country**				
	Croatia	400	78	478
	Poland	225	70	295
	Finland	254	21	275
Total		879	169	1048

### Correlation Between Age and Internet Addiction

There was a statistically significant correlation between age and the level of Internet addiction (χ^2^_6_=19.7; n=919; *P*=.003). Using the contingency table, we calculated that the percentage of moderate and serious addicts increased simultaneously with the participants’ age as follows: youngest (aged 11-12 years), 6%; slightly older (aged 13-14 years), 12%; older (aged 15-16 years), 19%; and the oldest (aged 17-18 years), 13%. The results showed a correlation between age and Internet addiction (χ^2^_3_= 13.5; n=919; *P*=.004). This correlation was further broken down by gender, country, and the purpose of Internet use. Thus, we could determine whether it was the male or female demographic that contributed to the correlation between age and Internet addiction. The same analysis was applied to the remaining two independent variables—country of residence and the purpose of Internet use. Due to the preciseness of tests, Internet addiction was expressed as normal addiction or moderate and serious addiction. Male participants and those who used the Internet mostly for entertainment purposes contributed the most to the correlation between the age of adolescents and Internet addiction (see Table 5).

**Table 5:** Contingency table (4 × 2 format) showing correlation between age of adolescent participants and Internet addiction.

Variables	Analyzed group	n	χ^2^	df	*P*
Age of adolescents (4 groups) Internet addiction (2 groups)	Male participants	469	16.9	3	.001
Age of adolescents (4 groups) Internet addiction (2 groups)	Female participants	447	0.5	3	.93
Age of adolescents (4 groups) Internet addiction (2 groups)	Participants from Croatia	397	5.8	3	.12
Age of adolescents (4 groups) Internet addiction (2 groups)	Participants from Poland	270	3.8	3	.29
Age of adolescents (4 groups) Internet addiction (2 groups)	Participants from Finland	252	1.1	3	.81
Age of adolescents (4 groups) Internet addiction (2 groups)	Participants who used the Internet for school/work	145	7.4	3	.06
Age of adolescents (4 groups) Internet addiction (2 groups)	Participants who used the Internet for entertainment	760	8.4	3	.04

In a correlation analysis, age was considered as a continuous independent variable and Internet addiction as a dependent ordinal variable. A nonparametric method was used to calculate the Spearman coefficient of correlation of .08 for n=1033 and with *P*=.01, showing that the correlation between the age of adolescents and Internet addiction was weak, but positive and statistically significant. We carried out a two-factor variance analysis. The dependent quantitative variable was Internet addiction (expressed in points); this variable was formed as a sum of scores of the answers to the 20 questions on Internet use. The first independent categorical variable was the purpose of Internet use (factor 1) with two modalities: school/work and entertainment. The second independent categorical variable was the age group (factor 2) with four modalities: 11-12, 13-14, 15-16, and 17-18 years.

### Influence of the Purpose of Internet Use on the Level of Internet Addiction

In describing the analysis data obtained through the described model, the Levene test of variance equality has to be mentioned first, as it has established that variances were not homogenous (*P*<.001) for the analyzed sample of participants.

The obtained results (see Table 6) showed that there was no significant influence of the purpose of Internet use on the level of Internet addiction, disregarding the age of participants (*P*=.22). In addition, there was a statistically important influence of the age of participants on the level of Internet addiction, disregarding the purpose of Internet use (*P*<.001). Finally, there was a statistically significant interaction between the purpose of Internet use and age regarding the level of Internet addiction (*P*=.001).

### Age and the Level of Internet Addiction

Comparing the eight mean values among themselves, we are able to determine the lowest and the highest level of Internet addiction. The lowest level of Internet addiction was found among the youngest age group who used the Internet for school, whereas adolescents between the ages of 15 and 16 years who used the Internet for school had the highest level of Internet addiction (see Table 7).

A single-factor variance analysis (*F* test) was used to compare the average values of Internet addiction in adolescents of different ages; points for average values were calculated based on the answers to the 20 questions in the questionnaire (see Table 8).

The average overall score was 37.8 (SD 12.3). There was a statistically significant difference between the four averages (*F*_3_=14.5; n=919; *P*<.001). A post hoc test according to Bonferroni yielded five statistically significant *P* values out of six possible values (see Table 9).

**Figure 2:**
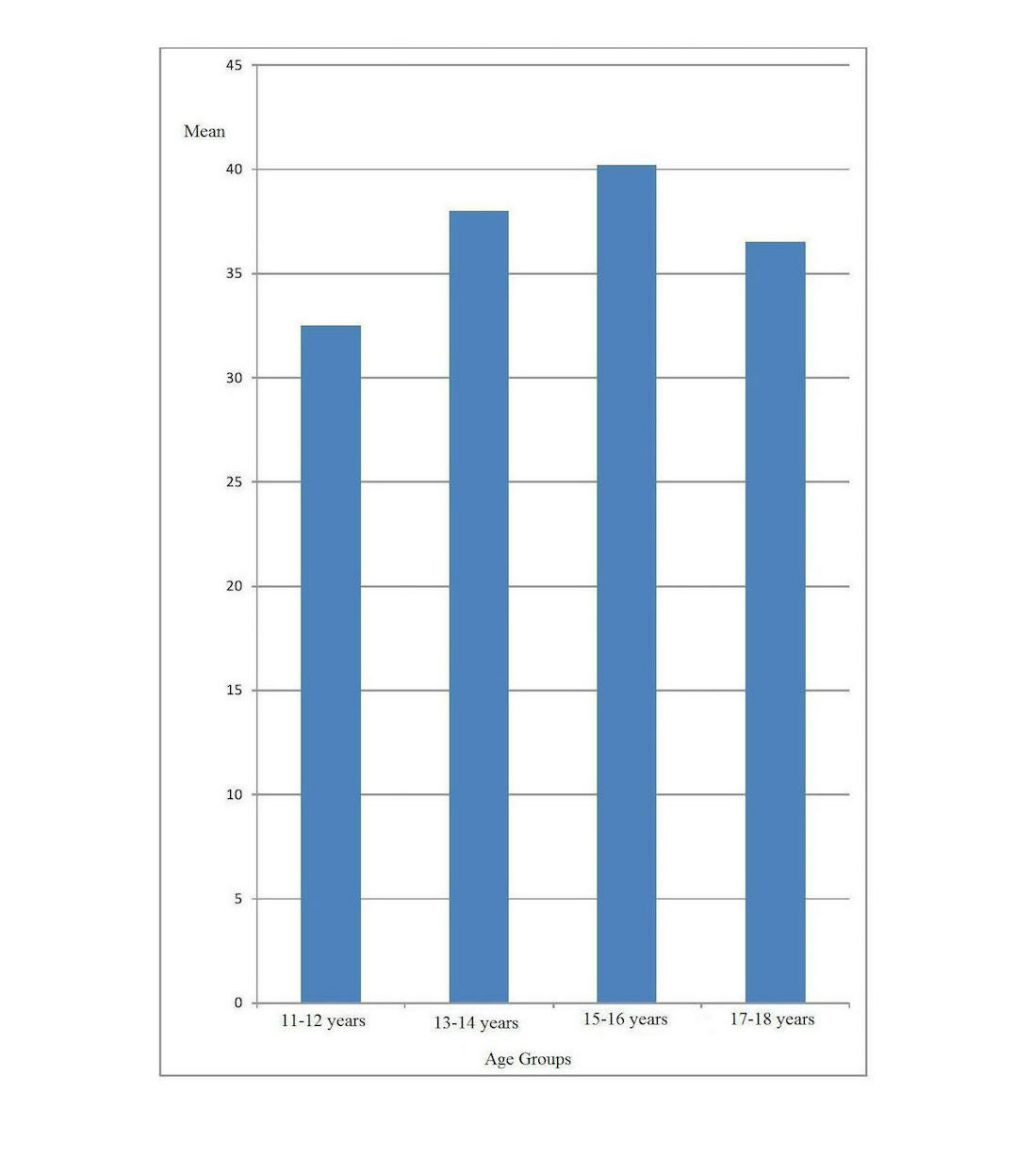
Comparison of the average values (points) of Internet addiction in adolescent participants of different age.

Adolescents aged 15-16 years who also used the Internet for school had the highest level of Internet addiction (see Figure 2).

**Table 6:** The analysis data obtained through the described model.

Source variation	The sum of squares	df	The middle square	*F*	*P*
Factor 1: the purpose of Internet use	220.152	1	220.152	1.54	.22
Factor 2: age	6686.400	3	2228.800	15.61	<.001
Interaction: the purpose of Internet use x age	2286.327	3	762.109	5.33	.001

**Table 7:** Summary results of descriptive statistical analysis with analysis of variance.

Age group (years)	Purpose of Internet use	Mean (SD)
**11-12**		
	School/work	32.5 (9.5)
	Entertainment	32.8 (9.4)
**13-14**		
	School/work	34.8 (9.1)
	Entertainment	38.2 (10.9)
**15-16**		
	School/work	46.5 (20.2)
	Entertainment	39.3 (11.9)
**17-18**		
	School/work	38.2 (11.1)
	Entertainment	35.9 (11.9)

**Table 8:** Results of the *F* test comparing the arithmetic means of Internet addiction (summary variable).

Age group (years)	n	Internet addiction, mean (SD)	*F*	*P*
11-12	115	32.4 (9.5)		
13-14	267	37.6 (10.7)		
15-16	323	40.4 (13.9)		
17-18	205	36.4 (12.0)	14.46	.001^a^

^a^Statistical significance at 0.1%.

**Table 9:** Results of the Bonferroni post hoc test (*P* values).

Age group (years)	Age group (years), *P*		
	13-14	15-16	17-18
11-12	.001^a^	<.001^a^	.03^b^
13-14		.004^b^	>.99
15-16			.001^a^

^a^Statistical significance at 0.1%.

^b^Statistical significance at 5%.

## Discussion

### Principal Findings

The objective of our study was to investigate if there were differences among adolescents in Croatia, Poland, and Finland regarding the purpose of their Internet use with respect to their age subgroup and gender. Our findings showed that a majority of adolescents used the Internet for entertainment and only one-sixth of them used it for school/work. Female adolescents used the Internet for school/work significantly more than did their male counterparts. The use of the Internet for school was most frequent among Polish adolescents, followed by Croatian adolescents, and least common among Finnish adolescents. Lenhart and Madden found similar results in their study, reporting that male adolescents in America use the Internet for functional and entertainment activities much more than female adolescents who use it for educational and social activities to a much higher degree [28]. Furthermore, similar results were also obtained by Tsai and Lin [29]. They concluded that Taiwanese boys use the Internet to elevate their mood, while Taiwanese girls have a more practical view of the Internet. Programs and activities on the Internet offer younger generations new dimensions of social activities, so the use of the Internet can expand and reinforce their connection with friends and colleagues [29]. One study reported that some adolescents were so preoccupied with their activities on the Internet that they were displaying signs of addiction [30]. In accordance with the correlations of risky forms of behavior and the developmental stages of adolescence, it seems that different stages of adolescence show a different percentage of Internet addicts. We also found a weak but positive correlation between the age of adolescents and Internet addiction. The greatest contributors to this correlation were male adolescents and those who used the Internet predominately for entertainment. Those aged 15-16 years demonstrated the highest degree of addiction, possibly because at this age they have achieved a greater level of independence and their free time and social activities are less controlled by their parents. Online communication has a strong influence on the developmental stages of adolescence. Adolescents share their experience through new forms of communication; they seek their own position within a group and report their friends as being a great source of social support, even greater than their parents [31]. According to our results, there is no statistically significant influence of the purpose of Internet use on the level of Internet addiction, while there is a statistically significant influence of the age of adolescents on the level of Internet addiction. In addition, there is a statistically significant interaction between the purpose of Internet use and age with regard to the level of Internet addiction. We established that the lowest level of addiction occurred among the youngest group aged 11-12 years who use the Internet for school. They were also the ones who used the Internet for entertainment the least. The highest level of Internet addiction was found among the adolescents aged 15-16 years. They used the Internet for school as well as for entertainment more than other age groups of adolescents in our study. Those aged 13-14 years used the Internet more for entertainment and less for school, similar to those in the 17-18-year-old age group. Our results also showed that Internet addiction was highest among the adolescents aged 15-16 years.

### Limitations

Our study did not manage to clarify the relationship between the different developmental phases of adolescence and the growing number of Internet addicts. Another limitation of this study is that we have no specific clinical criteria to determine which children aged 15-16 years are the most sensitive and most vulnerable to Internet addiction. Thus, the current findings increase our understanding of the relationships between Internet addiction and different developmental phases of adolescence.

### Conclusions

The primary purpose of Internet use among the 1078 participants was entertainment (905/1078, 84.00%) while the secondary purpose was school/work (172/1078, 16.00%). A total of 20.0% (105/525) of female adolescents used the Internet for school/work, which was significantly more than the males (64/534, 12.0%). Croatian participants (78/486, 16.0%) used the Internet for school/work significantly less than did the Polish participants (71/296, 24.0%); the Finnish participants used it for school/work the least (24/296, 8.0%). The percentage of moderate and serious Internet addicts increased with age. Most adolescent Internet addicts were in the middle stage of adolescence (ie, the 15-16-year-old age group). Furthermore, the purpose of Internet use had no effect on the level of Internet addiction. Age was a significant predictor of the level of Internet addiction as was the interaction between the purpose of Internet use and age. After comparing the eight mean values between themselves, we learned that the level of Internet addiction was the lowest among the youngest group (11-12 years) that used the Internet for school. They also had the lowest rate of Internet use for entertainment. The highest level of Internet addiction was found among the group of 15-16-year-olds. This group had the highest rate of Internet use for school, but also for entertainment. In the 13-14-year-old age group, 38.2% (116/303) of adolescents used the Internet for school and 34.2% (104/303) for entertainment. In the 17-18-year-old age group, 38.2% (91/237) of adolescents used the Internet for school/work and 35.9% (85/237) used it for entertainment.

## Abbreviations

DSM: Diagnostic and Statistical Manual of Mental Disorders
IAT: Internet Addiction Test
